# Causal links between serotonin dynamics and cued fear learning: evidence from experimental studies

**DOI:** 10.3389/fncom.2026.1750926

**Published:** 2026-01-23

**Authors:** Afarin Badripour, Taegon Kim

**Affiliations:** 1 Brain Science Institute, Korea Institute of Science and Technology (KIST), Seoul, Republic of Korea; 2 Division of Bio-Medical Science and Technology, KIST School, Korea University of Science and Technology (UST), Seoul, Republic of Korea

**Keywords:** cued fear conditioning and extinction, dorsal raphe nuclei (DRN), selective serotonin reuptake inhibitor (SSRI), serotonin, serotonin transporter (5-HTT/SERT)

## Abstract

Serotonin is thought to regulate emotional learning and memory, but there remains much to be explored regarding its causal role in cued fear conditioning and extinction (CFC-E). Recent *in vivo* recording of dorsal raphe nucleus serotonin neuronal activity during CFC-E paradigm showed that the time course of serotonin level includes both rapid responses to conditioned and unconditioned stimuli and a slowly accumulating component that spans inter-trial intervals and reverses during extinction. By reviewing the studies that directly link the fear expression during CFC-E to the acute or chronic perturbations of serotonin dynamics at the organism level or within specific brain areas via pharmacological, genetic, and projection-specific manipulations, we argue that theoretical models defining the causal role of serotonin must incorporate continuous-time serotonin dynamics.

## Introduction

Defining the general role of serotonin is tempting yet intricate due to the inherent complexity of the serotonin system of a brain arisen from the widespread projection of raphe serotonergic neurons (Ren et al., 2019) and the diversity of serotonin receptor subtypes (Roth et al., 2000; Salvan et al., 2023). Theoretical approaches have leveraged reinforcement learning (RL) frameworks to interpret experimental observations on the correlation between serotonin-related signals and behavior, proposing that serotonin may act as a discounting factor (Doya, 2002), a reward-related parameter (Liu et al., 2020), a signal for uncertainty (Grossman et al., 2022), a broadcast of emotional salience (Paquelet et al., 2022), an opponent signal to dopamine (Daw et al., 2002), and so on. A recent study integrated these perspectives under the framework of prospective coding (Harkin et al., 2025). However, causal relationships between extracellular serotonin levels and behavior remain unclear, highlighting the need for mechanistic models that specify how the local or global serotonin level modulates behavior. Beginning with a specific task would be a good choice for constructing such a model, not just for simplicity but also for correct understanding. Task-specificity emphasizes two essential viewpoints. First, because the experimental paradigm unfolds over a continuous-time, the temporal dynamics of serotonin, rather than its average level, must be incorporated into the framework. Second, a task constrains the relevant neural circuits and brain regions, shaping interpretations of function of serotonin in a spatially specific manner. Therefore, to build a theoretical framework that bridges cognitive-level and physiological-level understanding of the serotonin system, it is necessary to begin with specifying the task and to incorporate the spatiotemporal dynamics of the extracellular serotonin level.

In this review, we focus on the cued fear conditioning and extinction (CFC-E) paradigm. Although the CFC-E protocol is well established and the underlying neural circuits have been intensively investigated, the dynamical role of serotonin in CFC-E has not scrutinized sufficiently. We first summarize the study that measured endogenous temporal dynamics of serotonin throughout CFC-E, particularly using the activity of serotonergic neurons in dorsal raphe nuclei (DRN). We then review “intervention-type” studies that are assumed to directly perturb the extracellular serotonin level via pharmacological, optogenetic, chemogenetic manipulation, or genetically engineered animal, and measured fear responses under CFC-E framework.

The studies reviewed here were systematically identified from an extensive list generated by a Pubmed search using a combination of the keywords, 5-HT and CFC-E, and were limited to the studies published since 2000. To maintain a focused scope, we excluded studies that examined only general anxiety-like behavior without associative learning, those that involved only contextual fear learning other without cued learning, and those that manipulated 5-HT receptors instead of directly altering extracellular serotonin.

## Temporal dynamics of extracellular serotonin during CFC-E

To directly link extracellular serotonin levels to behavioral output, monitoring the dynamics of brain-wide extracellular serotonin level would be ideal, although this remains technically challenging. As an alternative, the activity of raphe nuclei (RN) serotonergic neurons can serve as a useful proxy, given that the primary source of serotonin of a brain originates from RN serotonergic neurons, predominantly located in DRN and median RN (MRN). A recent study employing *in vivo* fiber photometry of genetically encoded calcium indicator monitored DRN serotonergic neuronal activity across the continuous-time of CFC-E (Sengupta and Holmes, 2019). In this study, DRN serotonergic neurons were responded rapidly at the time of the unconditioned stimulus (US; footshock). With repeated CS-US pairings, the initially sharp calcium transient aligned to US broadened, and its onset gradually shifted toward the conditioned stimulus (CS) onset, consistent with other recent studies (Grossman et al., 2022; Harkin et al., 2025; Liu et al., 2020). Notably, a slowly elevating activity component emerged during the inter-trial pauses between conditioning trials and gradually decayed by repeating extinction trials.

Although MRN serotonergic neurons, the other source of serotonin, were also activated by aversive stimuli, their overall calcium signal elevation was markedly weaker than that observed in DRN, and CS-US pairing dependent onset shifts were not detected (Kawai et al., 2022; Morishita et al., 2026). Moreover, the target brain areas of MRN serotonergic neuronal projections, such as medial habenula, ventral hippocampus, and interpeduncular nuclei, were distinct from those of the DRN, which supplies serotonin to brain areas critically involved in CFC-E, such as the amygdala (Kawai et al., 2022; Vertes et al., 1999). Accordingly, it is reasonable to assume the DRN as the primary source of serotonin during CFC-E, in contrast to contextual fear learning, in which MRN contributions should not be ignored (Andrade et al., 2013).

CS–US pairing–related fast (millisecond–second scale) calcium signals reflecting the activity dynamics of DRN serotonergic neurons appear to reasonably represent extracellular serotonin levels. This interpretation is supported by direct measurements of local extracellular serotonin concentration changes in the basolateral amygdala (BLA) and medial prefrontal cortex (mPFC) during CFC using iSeroSnFR (Unger et al., 2020), a genetically encoded serotonin indicator (GESI). Although serotonin dynamics during CFC-E have not yet been directly monitored, other GESIs, such as GPCR-Activation-Based 5-HT sensors (GRAB_5-HT_) (Deng et al., 2024; Wan et al., 2021) or sDarken (Kubitschke et al., 2022), may provide more definitive confirmation. In parallel, despite its poor temporal resolution, *in vivo* microdialysis (Zhao and Piatkevich, 2023) remains a useful method for assessing longer-term changes in local extracellular serotonin levels. Indeed, slowly elevating (minute scale) inter-trial calcium signal may also serve as a reliable proxy not only for the activity of DRN serotonergic neurons but also for extracellular serotonin levels, considering a study showing an overall elevation and long-term retention of extracellular serotonin levels in the amygdala following CS-US pairing as measured by *in vivo* microdialysis (Yokoyama et al., 2005). Additionally, because repetition of US without pairing with CS did not elicit a significant increase in serotonin levels on this time scale (Zanoveli et al., 2009), the slow component of serotonin dynamics observed during CFC-E may be conjectured to reflect an associative learning-related process rather than a nonspecific response to aversive stimulation.

## Intervention studies

### Acute intervention at the organism level

Straightforward approaches have been the systemic administration of selective serotonin reuptake inhibitors (SSRIs) via intraperitoneal (IP) injection, which are assumed to influence the whole brain (Burghardt et al., 2007; Burghardt et al., 2004; Payet et al., 2023; Ravinder et al., 2013). Acute SSRI treatment, especially citalopram, 1 h before conditioning produced faster acquisition of fear memory. This was indicated by higher freezing levels during the training session, and higher fear expression with longer memory retention during test period compared with saline (Burghardt et al., 2004). When fluoxetine was used instead of citalopram in a similar paradigm, the effects were weaker (Ravinder et al., 2013). In this case, freezing showed a transient increase in the middle of the conditioning sessions, but there was no significant overall difference across the entire conditioning period. During testing period, fear expression remained high but without clearly prolonged retention of fear memory. Another study also reported higher fear expression following fluoxetine during recall phase, though without revealing the temporal dynamics of memory retention (Marcinkiewcz et al., 2016). Difference in the magnitude of SSRI effects may stem from distinct pharmacokinetics (Hiemke and Härtter, 2000) or different actions on receptors (Ni and Miledi, 1997). Although further work is needed, acute SSRI treatment shortly before training appears to exert an anxiogenic effect. Interestingly, a recent study reported that fluoxetine reduced fear expression during conditioning, which is opposite to the earlier reports, yet fear memory during testing was stronger and longer-lasting than in vehicle-treated animals (Payet et al., 2023). This discrepancy may be related to the higher fluoxetine dose and the shorter interval between injection and conditioning. Similarly, citalopram or fluoxetine injection 1 h before extinction period increased fear expression during testing (Burghardt et al., 2007). A notable finding is that the heightened fear expression following acute treatment of citalopram was blocked by 5-HT2C receptor antagonist, whereas a 5-HT3 receptor antagonist had no effect. This result is potentially consistent with the weaker effect from fluoxetine, which is known to inhibit 5-HT2C receptors (Ni and Miledi, 1997).

### Chronic intervention at the organism level

Long-term (21–22 days) SSRI administration prior to habituation has been used as a pharmacological model of chronic modulation of the serotonin system at the organism level (Burghardt et al., 2004; Deschaux et al., 2011; Karpova et al., 2011). In contrast to the acute treatment in the same study, chronic citalopram did not alter conditioning, but reduced fear expression compared to saline (Burghardt et al., 2004). Chronic fluoxetine before conditioning did not produce significant differences during conditioning or extinction learning (Deschaux et al., 2011; Karpova et al., 2011). Chronic SSRI treatment has also been applied after conditioning but before extinction period (Burghardt et al., 2013; Deschaux et al., 2011; Karpova et al., 2011). Under this condition, chronic citalopram markedly slowed fear extinction compared to control, indicating impaired of extinction learning (Burghardt et al., 2013), whereas subchronic treatment (9 days) had no effect, implying a threshold duration for altering extinction. By contrast, fluoxetine treatment did not exhibit notable effects during extinction period (Deschaux et al., 2011; Karpova et al., 2011).

Unlike acute interventions, chronic manipulations also include genetic approaches. Given SSRIs target the serotonin transporter (SERT/5-HTT) (Bowman and Daws, 2019), both global knock-out (KO) and overexpression (OE) models have been studied.

5-HTT KO mice showed no difference from wild type in CFC-E (Wellman et al., 2007), whereas KO rats showed elevated fear expression during acquisition and extinction (Johnson et al., 2019). Conversely, 5-HTT OE mice exhibited reduced conditioned fear response during testing period (Barkus et al., 2014; Line et al., 2014). Considering the anxiolytic-like effect of the acute 5-HT2C receptor antagonism (Burghardt et al., 2007), organism-level effects of 5-HT2C receptor deletion are also of particular interest. Indeed, 5-HT2C receptor KO mice displayed significantly reduced fear expression during extinction period, reflecting facilitated extinction learning (Bouchekioua et al., 2022; Süß et al., 2022). As discussed above for fluoxetine, which inhibits 5-HT2C receptors, the minimal effects of fluoxetine may be explained by such anxiolytic-like consequences of 5-HT2C receptor loss of function.

### Intervention on specific brain area: (extended) amygdala and cortex

Local fluoxetine infusion before conditioning into either the central amygdala (CeA) or bed nucleus of the stria terminalis (BNST) revealed that only BNST infusion enhanced fear expression during testing period (Ravinder et al., 2013). On the other hand, although IP injection of citalopram before conditioning increased fear expression and prolonged memory retention (Burghardt et al., 2004), local infusion of the 5-HT2C receptor antagonist RS-102221 into the BNST after systemic SSRI treatment and before conditioning abolished this effect (Pelrine et al., 2016). Similarly, the elevated fear expression observed in SERT KO rats was reduced by local infusion of a 5-HT1A antagonist into BLA (Johnson et al., 2019). The 5-HT2C receptor antagonist SB242084, infused into BLA, produced an anxiolytic effect only in animals that had been stressed before conditioning (Baratta et al., 2016).

Optogenetic approach using SERT-Cre mice has been used to bidirectionally control the serotonin release in defined projection targets. Optogenetic excitation of DRN neurons projecting to the basal amygdala (BA) via light stimulated channelrhodopsin-2 (ChR2) during conditioning enhanced fear expression during conditioning and extinction, whereas light stimulation during extinction period did not alter extinction dynamics. Photoinhibition of the same projection from DRN to BA via light-activated chloride channel, iC++ (Berndt et al., 2016), the improved variant of iC1C2 (Berndt et al., 2014) (inhibitory version of channelrhodopsin chimera C1C2), during conditioning abolished the enhancement of fear response during conditioning and reversed the effect during extinction, resulting in reduced fear expression. However, the conditioned fear response at testing was not distinctive between control and photoinhibited group (Sengupta and Holmes, 2019). When DRN neurons projecting to the BNST were excited via ChR2 during conditioning, fear expression during test period was higher in the light-stimulation group than in controls (Marcinkiewcz et al., 2016), which is consistent with the earlier finding that local fluoxetine infusion into BNST increased fear expression during testing (Ravinder et al., 2013). Chemogenetic activation of DRN serotonergic neurons projecting to the CeA also elevated conditioned fear response during conditioning and extinction, whereas conditional KO of the enzyme protein, tryptophan hydroxylase 2 (Tph2) which is necessary for synthesizing serotonin in the same neuron group had no detectable effect (Ren et al., 2018). Reducing serotonergic fiber innervation of the lateral amygdala (LA) by local infusion of the neurotoxin, 5′,7′-dihydroxytryptamine (5,7-DHT) increased the amplitude of the fear-potentiated startle, indicating higher fear expression (Tran et al., 2013). When 5,7-DHT was infused into a broader region encompassing both BLA and LA, fear memory formation was severely impaired (Johnson et al., 2015). Although several amygdala subnuclei remain untested, the overall pattern suggests that higher serotonin levels in the amygdala (especially BLA) and BNST are associated with enhanced fear expression.

In contrast, drug infusion perturbing serotonin system into mPFC, another key region in CFC-E, or the other types of intervention targeting mPFC directly combined with CFC-E paradigm, are scarce. Notably, neither chemogenetic activation nor conditional Tph2 KO of DRN neuron group projecting to orbitofrontal cortex (OFC) altered fear response during conditioning or extinction (Ren et al., 2018).

## Discussion

In this review, we limited the survey to studies that directly compared perturbed extracellular serotonin levels with fear responses measured within the CFC-E paradigm (Table 1). In these studies, freezing—one of the passive defensive responses—was used as a measurable index of fear expression. However, brain-wide alterations in serotonin system are also capable of modulating general motor function, such as locomotion, independently of the neural circuits specifically involved in CFC-E (Dayan and Huys, 2009). To ensure that changes in freezing behavior reflect genuine fear-related immobility rather than nonspecific motor impairments, basic motor function assays, including open-field test and home-cage activity monitoring, have been employed as control measures in these experiments. Alternatively, suppression of an ongoing operant behavior—namely conditioned suppression—can be used to minimize potential confounds arising from motor function alterations when assessing fear expression (Bouchekioua et al., 2022).

**Table 1:** Summary table of the collection reviewed here.

Pharmacological										
Acute/chronic	Drug	Experimental paradigm				*C*	*E*	*R*	Note	References
Acute	Citalopram		*C*		*E*	↑	↑			Burghardt et al. (2004)
	Fluoxetine		*C*		*E*	–	↑			Ravinder et al. (2013)
	Fluoxetine		*C*		*E*	–	↑		No temporal info	Marcinkiewcz et al. (2016)
	Fluoxetine		*C*		*E*	↓	↑			Payet et al. (2023)
	Citalopram		*C*		*E*	–	↑		Effect abolished by 5-HT2CR antagonist	Burghardt et al. (2007)
	Fluoxetine		*C*		*E*	–	↑			
Chronic	Citalopram		*C*		*E*	–	↓			Burghardt et al. (2004)
	Fluoxetine		*C*		*E*	–	–			Deschaux et al. (2011)
	Fluoxetine		*C*		*E*	–	–	↓		
	Fluoxetine		*C*		*E*	–	–	↓	No temporal info	Karpova et al. (2011)
	Fluoxetine		*C*		*E*	–	–	↓	No temporal info, 14 days of treatment	
	Citalopram		*C*		*E*	–	↑			Burghardt et al. (2013)

Genetically modified					
Genetic modification	*C*	*E*	*R*	Note	References
SERT knock-out	–	–	↑	C57BL/6J mice	Wellman et al. (2007)
SERT knock-out	↑	↑		Wistar rats	Johnson et al. (2019)
SERT knock-out (hetero)	↑	↑		Wistar rats, lower-level expression of SERT	
SERT overexpression	–	↓		No temporal info	Barkus et al. (2014)
SERT overexpression	–	↓		No temporal info	Line et al. (2014)

Perturbation on specific brain area						
Target area	Method	*C*	*E*	*R*	Note	References
CeA	Fluoxetine, local infusion	–	–			Ravinder et al. (2013)
BNST	Fluoxetine, local infusion	–	↑			
BA	Optogenetic excitation of DRN neurons	↑	↑	↑	Photoexcitation during blue shading period	Sengupta and Holmes (2019)
		–	–	↑		
	Optogenetic inhibition of DRN neurons	–	↓	↓		
		–	–	↓		
BNST	Optogenetic excitation of DRN neurons	–	↑		Photoexcitation before conditioning	Marcinkiewcz et al. (2016)
CeA	Chemogenetic activation of DRN neurons	↑	↑		Chemogenetic activation before conditioning	Ren et al. (2018)
OFC		–	–			
CeA	Conditional Tph2 KO	–	–			
OFC		–	–			
LA	5,7-DHT, local infusion	–	↑		Fear-potentiated startle	Tran et al. (2013)
LA, BLA	5,7-DHT, local infusion	↓	↓			Johnson et al. (2015)

Green shading in pharmacology part is the time of treatment. Light green: acute treatment, dark green: chronic treatment (for 21–22 days). In the part of perturbation on specific brain area, light blue shading stands for optogenetic stimulation. *C*, conditioning period; *E*, extinction period; *R*, fear renewal period. Upward arrow (↑): increased conditional freezing response, downward arrow (↓): decreased conditional freezing response, dash (–): no significant effect.

At the organism level, acute SSRI treatment tends to increase fear expression and prolong memory retention. Considering both the pharmacological target of SSRIs (SERT) and the two components of endogenous serotonin dynamics (one rapidly responding to stimuli and one slowly accumulating and clearing), acute SSRI effects can be interpreted as a “blurring” of rapid serotonin transients responding to US or paired CS and an enhancement of the slow component. Specifically, acute SSRIs likely prolong and amplify the slow accumulation of serotonin during conditioning and delay its clearance during extinction. Under this view, rapid, CS-locked serotonin transients may simply broadcast the presence of a predictive signal to retrieve the associated US memory, without carrying the information of memory strength. In contrast, the slowly accumulated component of extracellular serotonin, persisting during inter-trial intervals, may maintain the CS-US association and hinder extinction. Excessively elevated serotonin levels could therefore represent a parameter that scales the obstruction of the association learning between cue and safety (extinction learning), consistent with the idea that successful extinction depends primarily on extinction learning early in the extinction session rather than on direct erasure of the original fear memory (Liu et al., 2024; Myers et al., 2006).

Although chronic treatment of fluoxetine, whether applied before conditioning or before extinction, showed little effect on conditioning and extinction, studies that additionally assessed fear renewal revealed that both pre- and post-conditioning chronic fluoxetine treatments aided to prevent the renewal of fear memory after extinction (Deschaux et al., 2011; Karpova et al., 2011). The same paradigm using citalopram, however, introduces a conundrum: chronic pre-conditioning citalopram reduced fear expression during extinction, whereas chronic pre-extinction citalopram increased fear expression and impaired extinction learning (Burghardt et al., 2013; Burghardt et al., 2004). Reconsidering the endogenous dynamics of serotonin release (Sengupta and Holmes, 2019), chronic SSRI treatment may already saturate the slowly varying component, thereby altering how new learning is integrated. Given the implied role of 5-HT2C receptors in promoting high fear expression, differences in extinction learning between fluoxetine and citalopram treatments are unlikely to be explained solely by the magnitude of slowly varying component of serotonin dynamics. There are two essential mechanisms that may contribute to such discrepancy from different types of SSRIs: pharmacokinetics and off-target actions. First, SSRIs differ in both the rate and extent of SERT engagement. Citalopram exhibits high affinity for SERT and produces substantial transporter occupancy within hours of administration (Finnema et al., 2015; Meyer et al., 2004). In contrast, fluoxetine and its active metabolite norfluoxetine accumulate more slowly due to their long half-lives, resulting in a gradual buildup of SERT blockade (Hiemke and Härtter, 2000; Lucas, 1992). Second, SSRIs differ in their off-target actions on serotonin receptors. In particular, fluoxetine shows appreciable affinity for 5-HT2C receptors and inhibitory effect on those (Ni and Miledi, 1997), which influence amygdala and prefrontal circuits that are critically involved in conditioned fear expression and extinction (Millan et al., 2003). Additionally, considering that SERT occupancies following citalopram (or escitalopram) administration varies across brain regions (Baldinger et al., 2014), the effect heterogeneity is likely to differ among SSRI types, leading to distinct regulation of neural circuits relevant to conditioned fear expression. Accordingly, future theoretical frameworks will need to capture the joint effects of SSRI type, heterogenous circuit-level modulation, regulation of the slow component of serotonin dynamics, extinction learning, and fear memory erasure.

In contrast to SSRI treatment, manipulations that presumably reduce extracellular serotonin levels (e.g., SERT/5-HTT overexpression) tend to yield reduced fear expression during extinction, comparable to wild-type controls (Barkus et al., 2014; Line et al., 2014). This observation is broadly consistent with our rough speculation that high baseline serotonin obstructs extinction learning. Additionally, 5-HT2C receptor KO mice showed facilitated extinction, reflected by lower fear expression and faster extinction (Bouchekioua et al., 2022; Süß et al., 2022), further supporting the view that excessive serotonin levels, acting via 5-HT2C receptors, impede extinction learning.

Because DRN serotonergic neurons provide widespread innervation throughout the brain (Ren et al., 2019), perturbing fiber integrity or terminal release in specific regions can only offer partial insight into the global relationship between serotonin dynamics and behavior. However, once a theoretical framework identifies how cognitive variables (e.g., prediction error, uncertainty, or safety learning) map onto dynamical variables of serotonin (e.g., baseline level, transient amplitude, timescale of decay), an additional question arises: how do these system-level correspondences and causal relationships emerge from local “unit machines,” such as microcircuits shaped by serotonin in specific brain regions? Even within the limited set of studies, we reviewed that directly link local serotonin level to behavior, there is a positive correlation between organism-level effects of serotonin on behavior and the effects observed when serotonin is perturbed in BLA or BNST. In contrast, direct manipulations of serotonin dynamics in mPFC in the context of CFC-E remain largely unexplored, perhaps because the diversity of 5-HT receptor subtypes expressed there makes circuit-level interpretations challenging and elaborate the complexity.

To more firmly establish causal links between serotonin dynamics and fear responses derived from associative memory, more evidence is clearly needed. Because we argue that continuous-time serotonin dynamics across the entire CFC-E experiment are critical, future studies should provide raw behavioral data not only during tone-shock pairing or tone-alone trials but also during inter-trial intervals (Süß et al., 2022). In addition, experimental paradigms that combine multiple timescales of serotonin modulation are likely to be especially informative. For example, acute SSRI or neurotoxin treatment in genetically modified animals (such as 5-HTT OE or 5-HTT KO) could help disentangle shorter- versus longer-timescale effects of serotonin dynamics on behavior. Similarly, combining approaches that are effective on different timescales—acute pharmacology, chronic drug treatment, genetic manipulations, and circuit-specific opto/chemogenetic interventions—may guide the construction of theoretical frameworks capable of explaining what aspects of serotonin dynamics are genuinely causal for fear learning and extinction. From the perspective of RL, multiscale temporal dynamics of extracellular serotonin levels also suggest that defining role of serotonin by assigning a fixed correspondence to a single hyperparameter—such as discounting factor or uncertainty—is unlikely to be a promising approach. Thus, as a next step toward an integrative framework, RL models may benefit from incorporating time-dependent hyperparameters that exhibit multiple correspondences with serotonin dynamics across different timescales. Such a formulation would allow dynamic transitions between distinct learning regimes over time, e.g., between model-free and model-based RL.

## References

[ref1] Andrade T. G. Zangrossi H. Graeff F. G. (2013). The median raphe nucleus in anxiety revisited. J. Psychopharmacol. 27, 1107–1115. doi: 10.1177/0269881113499208, 23999409

[ref2] Baldinger P. Kranz G. S. Haeusler D. Savli M. Spies M. Philippe C. . (2014). Regional differences in SERT occupancy after acute and prolonged SSRI intake investigated by brain PET. NeuroImage 88, 252–262. doi: 10.1016/j.neuroimage.2013.10.002, 24121201

[ref3] Baratta M. V. Kodandaramaiah S. B. Monahan P. E. Yao J. Weber M. D. Lin P.-A. . (2016). Stress enables reinforcement-elicited serotonergic consolidation of fear memory. Biol. Psychiatry 79, 814–822. doi: 10.1016/j.biopsych.2015.06.025, 26248536 PMC4698247

[ref4] Barkus C. Line S. J. Huber A. Capitao L. Lima J. Jennings K. . (2014). Variation in serotonin transporter expression modulates fear-evoked hemodynamic responses and Theta-frequency neuronal oscillations in the amygdala. Biol. Psychiatry 75, 901–908. doi: 10.1016/j.biopsych.2013.09.003, 24120093 PMC4032572

[ref5] Berndt A. Lee S. Y. Ramakrishnan C. Deisseroth K. (2014). Structure-guided transformation of channelrhodopsin into a light-activated chloride channel. Science 344, 420–424. doi: 10.1126/science.1252367, 24763591 PMC4096039

[ref6] Berndt A. Lee S. Y. Wietek J. Ramakrishnan C. Steinberg E. E. Rashid A. J. . (2016). Structural foundations of optogenetics: determinants of channelrhodopsin ion selectivity. Proc. Natl. Acad. Sci. USA 113, 822–829. doi: 10.1073/pnas.1523341113, 26699459 PMC4743797

[ref7] Bouchekioua Y. Nebuka M. Sasamori H. Nishitani N. Sugiura C. Sato M. . (2022). Serotonin 5-HT2C receptor knockout in mice attenuates fear responses in contextual or cued but not compound context-cue fear conditioning. Transl. Psychiatry 12:58. doi: 10.1038/s41398-022-01815-2, 35145065 PMC8831648

[ref8] Bowman M. A. Daws L. C. (2019). Targeting serotonin transporters in the treatment of juvenile and adolescent depression. Front. Neurosci. 13: Article 156. doi: 10.3389/fnins.2019.00156 PMC6401641 30872996

[ref9] Burghardt N. S. Bush D. E. McEwen B. S. LeDoux J. E. (2007). Acute selective serotonin reuptake inhibitors increase conditioned fear expression: blockade with a 5-HT(2C) receptor antagonist. Biol. Psychiatry 62, 1111–1118. doi: 10.1016/j.biopsych.2006.11.023, 17524369 PMC2129095

[ref10] Burghardt N. S. Sigurdsson T. Gorman J. M. McEwen B. S. LeDoux J. E. (2013). Chronic antidepressant treatment impairs the Acquisition of Fear Extinction. Biol. Psychiatry 73, 1078–1086. doi: 10.1016/j.biopsych.2012.10.012, 23260230 PMC3610782

[ref11] Burghardt N. S. Sullivan G. M. McEwen B. S. Gorman J. M. LeDoux J. E. (2004). The selective serotonin reuptake inhibitor citalopram increases fear after acute treatment but reduces fear with chronic treatment: a comparison with tianeptine. Biol. Psychiatry 55, 1171–1178. doi: 10.1016/j.biopsych.2004.02.029, 15184036

[ref12] Daw N. D. Kakade S. Dayan P. (2002). Opponent interactions between serotonin and dopamine. Neural Netw. 15, 603–616. doi: 10.1016/S0893-6080(02)00052-7, 12371515

[ref13] Dayan P. Huys Q. J. (2009). Serotonin in affective control. Annu. Rev. Neurosci. 32, 95–126. doi: 10.1146/annurev.neuro.051508.135607, 19400722

[ref14] Deng F. Wan J. Li G. Dong H. Xia X. Wang Y. . (2024). Improved green and red GRAB sensors for monitoring spatiotemporal serotonin release in vivo. Nat. Methods 21, 692–702. doi: 10.1038/s41592-024-02188-8, 38443508 PMC11377854

[ref15] Deschaux O. Spennato G. Moreau J.-L. Garcia R. (2011). Chronic treatment with fluoxetine prevents the return of extinguished auditory-cued conditioned fear. Psychopharmacology 215, 231–237. doi: 10.1007/s00213-010-2134-y, 21181120

[ref16] Doya K. (2002). Metalearning and neuromodulation. Neural Netw. 15, 495–506. doi: 10.1016/S0893-6080(02)00044-8, 12371507

[ref17] Finnema S. J. Halldin C. Bang-Andersen B. Bundgaard C. Farde L. (2015). Serotonin transporter occupancy by escitalopram and citalopram in the non-human primate brain: a [(11)C]MADAM PET study. Psychopharmacology 232, 4159–4167. doi: 10.1007/s00213-015-3961-7, 25980484

[ref18] Grossman C. D. Bari B. A. Cohen J. Y. (2022). Serotonin neurons modulate learning rate through uncertainty. Curr. Biol. 32, 586–599. doi: 10.1016/j.cub.2021.12.006, 34936883 PMC8825708

[ref19] Harkin E. F. Grossman C. D. Cohen J. Y. Béïque J.-C. Naud R. (2025). A prospective code for value in the serotonin system. Nature 641, 952–959. doi: 10.1038/s41586-025-08731-7, 40140568

[ref20] Hiemke C. Härtter S. (2000). Pharmacokinetics of selective serotonin reuptake inhibitors. Pharmacol. Ther. 85, 11–28. doi: 10.1016/S0163-7258(99)00048-0, 10674711

[ref21] Johnson P. L. Molosh A. I. Federici L. M. Bernabe C. Haggerty D. Fitz S. D. . (2019). Assessment of fear and anxiety associated behaviors, physiology and neural circuits in rats with reduced serotonin transporter (SERT) levels. Transl. Psychiatry 9:33. doi: 10.1038/s41398-019-0368-y, 30670681 PMC6343029

[ref22] Johnson P. L. Molosh A. Fitz S. D. Arendt D. Deehan G. A. Federici L. M. . (2015). Pharmacological depletion of serotonin in the basolateral amygdala complex reduces anxiety and disrupts fear conditioning. Pharmacol. Biochem. Behav. 138, 174–179. doi: 10.1016/j.pbb.2015.09.021, 26476009 PMC4631224

[ref23] Karpova N. N. Pickenhagen A. Lindholm J. Tiraboschi E. Kulesskaya N. Agústsdóttir A. . (2011). Fear erasure in mice requires synergy between antidepressant drugs and extinction training. Science 334, 1731–1734. doi: 10.1126/science.1214592, 22194582 PMC3929964

[ref24] Kawai H. Bouchekioua Y. Nishitani N. Niitani K. Izumi S. Morishita H. . (2022). Median raphe serotonergic neurons projecting to the interpeduncular nucleus control preference and aversion. Nat. Commun. 13:7708. doi: 10.1038/s41467-022-35346-7, 36550097 PMC9780347

[ref25] Kubitschke M. Müller M. Wallhorn L. Pulin M. Mittag M. Pollok S. . (2022). Next generation genetically encoded fluorescent sensors for serotonin. Nat. Commun. 13:7525. doi: 10.1038/s41467-022-35200-w, 36473867 PMC9726753

[ref26] Line S. J. Barkus C. Rawlings N. Jennings K. McHugh S. Sharp T. . (2014). Reduced sensitivity to both positive and negative reinforcement in mice over-expressing the 5-hydroxytryptamine transporter. Eur. J. Neurosci. 40, 3735–3745. doi: 10.1111/ejn.12744, 25283165 PMC4737229

[ref27] Liu Z. Lin R. Luo M. (2020). Reward contributions to serotonergic functions. Annu. Rev. Neurosci. 43, 141–162. doi: 10.1146/annurev-neuro-093019-112252, 32640931

[ref28] Liu Y. Ye S. Li X.-N. Li W.-G. (2024). Memory trace for fear extinction: fragile yet reinforceable. Neurosci. Bull. 40, 777–794. doi: 10.1007/s12264-023-01129-3, 37812300 PMC11178705

[ref29] Lucas R. A. (1992). The human pharmacology of fluoxetine. Int. J. Obes. Relat. Metab. Disord. 16, S49–S54. 1338386

[ref30] Marcinkiewcz C. A. Mazzone C. M. D’Agostino G. Halladay L. R. Hardaway J. A. DiBerto J. F. . (2016). Serotonin engages an anxiety and fear-promoting circuit in the extended amygdala. Nature 537, 97–101. doi: 10.1038/nature19318, 27556938 PMC5124365

[ref31] Meyer J. H. Wilson A. A. Sagrati S. Hussey D. Carella A. Potter W. Z. . (2004). Serotonin transporter occupancy of five selective serotonin reuptake inhibitors at different doses: an [11C]DASB positron emission tomography study. Am. J. Psychiatry 161, 826–835. doi: 10.1176/appi.ajp.161.5.826, 15121647

[ref32] Millan M. J. Gobert A. Lejeune F. Dekeyne A. Newman-Tancredi A. Pasteau V. . (2003). The novel melatonin agonist agomelatine (S20098) is an antagonist at 5-hydroxytryptamine2C receptors, blockade of which enhances the activity of frontocortical dopaminergic and adrenergic pathways. J. Pharmacol. Exp. Ther. 306, 954–964. doi: 10.1124/jpet.103.051797 12750432

[ref33] Morishita H. Hori H. Kawai H. Shirakawa H. Hashimoto H. Nagayasu K. (2026). Delayed response of the median raphe serotonin neurons projecting to the ventral hippocampus to aversive stimuli. J. Pharmacol. Sci. 160, 91–96. doi: 10.1016/j.jphs.2025.12.003 41554598

[ref34] Myers K. M. Ressler K. J. Davis M. (2006). Different mechanisms of fear extinction dependent on length of time since fear acquisition. Learn. Mem. 13, 216–223. doi: 10.1101/lm.119806, 16585797 PMC1409828

[ref35] Ni Y. G. Miledi R. (1997). Blockage of 5HT2C serotonin receptors by fluoxetine (Prozac). Proc. Natl. Acad. Sci. USA 94, 2036–2040. 9050900 10.1073/pnas.94.5.2036 PMC20038

[ref36] Paquelet G. E. Carrion K. Lacefield C. O. Zhou P. Hen R. Miller B. R. (2022). Single-cell activity and network properties of dorsal raphe nucleus serotonin neurons during emotionally salient behaviors. Neuron 110, 2664–2679. doi: 10.1016/j.neuron.2022.05.015, 35700737 PMC9575686

[ref37] Payet J. M. Stevens L. Russo A. M. Jaehne E. J. van den Buuse M. Kent S. . (2023). The role of dorsal raphe nucleus serotonergic systems in emotional learning and memory in male BALB/c mice. Neuroscience 534, 1–15. doi: 10.1016/j.neuroscience.2023.10.003, 37852412

[ref38] Pelrine E. Pasik S. D. Bayat L. Goldschmiedt D. Bauer E. P. (2016). 5-HT2C receptors in the BNST are necessary for the enhancement of fear learning by selective serotonin reuptake inhibitors. Neurobiol. Learn. Mem. 136, 189–195. doi: 10.1016/j.nlm.2016.10.008, 27773594 PMC5126982

[ref39] Ravinder S. Burghardt N. S. Brodsky R. Bauer E. P. Chattarji S. (2013). A role for the extended amygdala in the fear-enhancing effects of acute selective serotonin reuptake inhibitor treatment. Transl. Psychiatry 3:e209. doi: 10.1038/tp.2012.137, 23321806 PMC3566718

[ref40] Ren J. Friedmann D. Xiong J. Liu C. D. Ferguson B. R. Weerakkody T. . (2018). Anatomically defined and functionally distinct dorsal raphe serotonin sub-systems. Cell 175, 472–487. doi: 10.1016/j.cell.2018.07.043, 30146164 PMC6173627

[ref41] Ren J. Isakova A. Friedmann D. Zeng J. Grutzner S. M. Pun A. . (2019). Single-cell transcriptomes and whole-brain projections of serotonin neurons in the mouse dorsal and median raphe nuclei. eLife 8:e49424. doi: 10.7554/eLife.49424, 31647409 PMC6812963

[ref42] Roth B. L. Lopez E. Patel S. Kroeze W. K. (2000). The multiplicity of serotonin receptors: uselessly diverse molecules or an embarrassment of riches? Neuroscientist 6, 252–262. doi: 10.1177/107385840000600408

[ref43] Salvan P. Fonseca M. Winkler A. M. Beauchamp A. Lerch J. P. Johansen-Berg H. (2023). Serotonin regulation of behavior via large-scale neuromodulation of serotonin receptor networks. Nat. Neurosci. 26, 53–63. doi: 10.1038/s41593-022-01213-3, 36522497 PMC9829536

[ref44] Sengupta A. Holmes A. (2019). A discrete dorsal raphe to basal amygdala 5-HT circuit calibrates aversive memory. Neuron 103, 489–505. doi: 10.1016/j.neuron.2019.05.029, 31204082 PMC6687558

[ref45] Süß S. T. Olbricht L. M. Herlitze S. Spoida K. (2022). Constitutive 5-HT2C receptor knock-out facilitates fear extinction through altered activity of a dorsal raphe-bed nucleus of the stria terminalis pathway. Transl. Psychiatry 12:487. doi: 10.1038/s41398-022-02252-x, 36402746 PMC9675804

[ref46] Tran L. Lasher B. K. Young K. A. Keele N. B. (2013). Depletion of serotonin in the basolateral amygdala elevates glutamate receptors and facilitates fear-potentiated startle. Transl. Psychiatry 3:e298. doi: 10.1038/tp.2013.66, 24002084 PMC3784761

[ref47] Unger E. K. Keller J. P. Altermatt M. Liang R. Matsui A. Dong C. . (2020). Directed evolution of a selective and sensitive serotonin sensor via machine learning. Cell 183, 1986–2002. doi: 10.1016/j.cell.2020.11.040, 33333022 PMC8025677

[ref48] Vertes R. P. Fortin W. J. Crane A. M. (1999). Projections of the median raphe nucleus in the rat. J. Comp. Neurol. 407, 555–582. doi: 10.1002/(SICI)1096-9861(19990517)407:4<555::AID-CNE7>3.0.CO;2-E, 10235645

[ref49] Wan J. Peng W. Li X. Qian T. Song K. Zeng J. . (2021). A genetically encoded sensor for measuring serotonin dynamics. Nat. Neurosci. 24, 746–752. doi: 10.1038/s41593-021-00823-7, 33821000 PMC8544647

[ref50] Wellman C. L. Izquierdo A. Garrett J. E. Martin K. P. Carroll J. Millstein R. . (2007). Impaired stress-coping and fear extinction and abnormal corticolimbic morphology in serotonin transporter knock-out mice. J. Neurosci. 27, 684–691. doi: 10.1523/jneurosci.4595-06.2007, 17234600 PMC6672805

[ref51] Yokoyama M. Suzuki E. Sato T. Maruta S. Watanabe S. Miyaoka H. (2005). Amygdalic levels of dopamine and serotonin rise upon exposure to conditioned fear stress without elevation of glutamate. Neurosci. Lett. 379, 37–41. doi: 10.1016/j.neulet.2004.12.047, 15814195

[ref52] Zanoveli J. M. Carvalho M. C. Cunha J. M. Brandão M. L. (2009). Extracellular serotonin level in the basolateral nucleus of the amygdala and dorsal periaqueductal gray under unconditioned and conditioned fear states: an in vivo microdialysis study. Brain Res. 1294, 106–115. doi: 10.1016/j.brainres.2009.07.074, 19646971

[ref53] Zhao S. Piatkevich K. D. (2023). Techniques for in vivo serotonin detection in the brain: state of the art. J. Neurochem. 166, 453–480. doi: 10.1111/jnc.15865, 37293767

